# CULTURAL DIFFERENCES IN MEDICATION BURDEN OF OLDER HOSPITALIZED PATIENTS IN MEDICAL UNITS

**DOI:** 10.1093/geroni/igae098.2913

**Published:** 2024-12-31

**Authors:** Juliana Smichenko, Ksenya Shulyaev, Efrat Shadmi, Nosaiba Rayan-Gharra, Nurit Gur-Yaish, Anna Zisberg

**Affiliations:** University of Haifa, Haifa, Hefa, Israel; University of Haifa, Haifa, Hefa, Israel; University of Haifa, Haifa, Hefa, Israel; University of Haifa, Haifa, Hefa, Israel; Oranim Academic College, Kiryat Tivon, HaZafon, Israel; University of Haifa, Haifa, Hefa, Israel

## Abstract

Cultural differences are significantly related to the management of illness and symptoms, particularly in hospitalized older adults where medications are often employed for health stabilization. This study aimed to examine cultural variations in medication burden, specifically focusing on sedative and anticholinergic medications, among older patients admitted to medical units. A secondary analysis was conducted on data from 672 cognitively intact older adults (age 65 and above) who were functionally independent. The cohort comprised 104 Israeli Arabs, 165 former-USSR immigrants, and 403 veteran Israeli Jews. Upon admission, socio-demographic, functional, and cognitive assessments were performed, alongside daily evaluations of symptom severity. Medical records provided data on comorbidities, illness severity, and medication prescriptions, allowing for the calculation of the Drug Burden Index (DBI) to assess medication burden. Multiple regression analysis was employed to predict medication burden, considering cultural group, sex, age, functional and cognitive status, symptom severity, illness severity, comorbidities, and length of hospital stay. The model demonstrated significance (F(10, 662)=11.647, p<.001, R2=.151), revealing that being Israeli Arab (t=-3.174, p=.002) or former-USSR immigrant (t=-2.157, p=.031) acted as protective factors against medication burden. Symptom severity, illness severity (t=-5.613, p<.001; t=4.102, p<.001), and comorbidities (t=3.775, p<.001) emerged as significant predictors of higher medication burden. These findings underscore the presence of cultural disparities in medication prescribing during hospitalization. Despite similar presentations of symptoms and medical statuses across cultural groups, certain cultural backgrounds are associated with lower medication burdens. Understanding and exploring patient-physician dynamics within cultural contexts are essential for optimizing treatment approaches in diverse patient populations.

